# The Role of Nrf2 in the Regulation of Mitochondrial Function and Ferroptosis in Pancreatic Cancer

**DOI:** 10.3390/antiox13060696

**Published:** 2024-06-06

**Authors:** Dinara Baiskhanova, Heiner Schäfer

**Affiliations:** Laboratory of Molecular Gastroenterology and Tumor Biology, Institute for Experimental Cancer Research, Christian-Albrechts-University of Kiel, 24105 Kiel, Germany; hschaef@1med.uni-kiel.de

**Keywords:** Nrf2, oxidative stress, antioxidant genes, mitochondria, ferroptosis, pancreatic cancer

## Abstract

The transcription factor nuclear factor erythroid 2-related factor 2 (Nrf2) represents the master regulator of the cellular antioxidant response and plays a critical role in tumorigenesis. This includes a preventive effect of Nrf2 on cell death through ferroptosis, which represents an essential mechanism of therapy resistance in malignant tumors, such as pancreatic ductal adenocarcinoma (PDAC) as one of the most aggressive and still incurable tumors. Addressing this issue, we provide an overview on Nrf2 mediated antioxidant response with particular emphasis on its effect on mitochondria as the organelle responsible for the execution of ferroptosis. We further outline how deregulated Nrf2 adds to the progression and therapy resistance of PDAC, especially with respect to the role of ferroptosis in anti-cancer drug mediated cell killing and how this is impaired by Nrf2 as an essential mechanism of drug resistance. Our review further discusses recent approaches for Nrf2 inhibition by natural and synthetic compounds to overcome drug resistance based on enhanced ferroptosis. Finally, we provide an outlook on therapeutic strategies based on Nrf2 inhibition combined with ferroptosis inducing drugs.

## 1. Introduction

Reactive oxygen species (ROS) are highly reactive intermediary oxygen metabolites that are produced during oxygen metabolism. Though playing a fundamental role in many cellular processes [1], a higher concentration level of ROS is detrimental if left uncompensated, a condition termed oxidative stress (OS). Consequently, the production of ROS that takes place intracellularly in mitochondria (along with the electron transfer in the respiratory chain) and in peroxisomes (along with β-oxidation of fatty acids and through peroxisomal oxidases) as well as intra- and extracellularly by membrane-bound cellular oxidases (NOX and DUOX) needs tight control [2]. Moreover, ROS levels are kept low by a battery of neutralizing and deactivating enzymes [3] such as catalase, superoxide dismutase, and glutathione peroxidase.

A master regulator of these ROS controlling events is the transcription factor—nuclear factor erythroid 2-related factor 2 (Nrf2). Under homeostatic conditions with well-balanced ROS levels, Nrf2 is bound by Kelch-like ECH-associated protein 1 (Keap1) in the cytoplasm and is forwarded to its proteasomal degradation [4,5,6,7,8]. During OS, Nrf2 degradation is prevented through several mechanisms affecting its interaction with Keap1. Instead, the Nrf2 protein accumulates in the cytoplasm, translocates then into the nucleus, and binds to the antioxidant response element (ARE) of various transcriptional target genes. These include a battery of enzymes for ROS detoxification and regulatory proteins responsible for other antioxidant processes in the cytoplasm as well as in mitochondria [5]. Besides antioxidant genes, Nrf2 targets include also proteasomal, metabolic, and pro-survival genes, making Nrf2 an essential regulator of cellular integrity and homeostasis. Thus, Nrf2 has a pivotal role in cytoprotection and its functional impairment manifests in many disorders, including respiratory [9], cardiovascular [10], renal [11], and neuronal diseases [12]. However, Nrf2 dependent cryoprotection is also an essential driver in tumorigenesis [6,7,8,13,14]. It is well established that malignant transformation is characterized by a greater resistance to OS caused by a sustained Nrf2 activity (see below). Thus, greater tolerated ROS levels not only account for mutational events but also lead to the metabolic adaptation of cancerous cells, their survival, angiogenesis, and metastatic spread [15]. This includes the protection of cancer cells from a variant of programmed cell-death known as ferroptosis (see below), which greatly accounts for the chemoresistance of many tumors [16].

Among those tumors characterized by a profound chemoresistance is pancreatic ductal adenocarcinoma (PDAC). This tumor is one of the most aggressive and fatal malignancies and ranks 4th in cancer-related deaths, with the highest prevalence rates observed in Western countries. It is expected that this highly malignant disease will be the second leading cause of cancer-related deaths by 2030, affecting a still growing number of patients in the US and Europe. The severity of the disease is mostly due to the late diagnosis and the lowest survival rate compared to other types of tumors, which gives rise to 5-year survival period (with resectable tumor) for only 12.5% of patients [17]. Around 80% of pancreatic cancer patients present with progressive disease and metastases, which makes it impracticable to cure them [18]. The majority (90%) of pancreatic tumors are represented by pancreatic ductal adenocarcinoma (PDAC) [19,20], which is characterized by an early metastatic spread and an exceptional resistance to conventional anti-cancer therapies currently available [19]. Thus, gemcitabine monotherapy or the combined treatment with folic acid, 5-FU, irinotecan, and oxaliplatin (FOLFIRINOX) [21,22,23] yield only a moderate improvement for most PDAC patients that are not eligible for curative resection [24]. The aggressiveness and chemoresistance of PDAC is particularly due to intrinsic and extrinsic mechanisms overcoming the deleterious effects of reactive oxygen species (ROS). Since an elevated ROS-stress essentially contributes to the cytotoxic effect of many anti-cancer drugs [25,26], including gemcitabine or the FOLFIRINOX treatment in PDAC [27], the already ROS-adapted cancer cells reveal greater resistance to such treatments. A better understanding of Nrf2 driven ROS protection and ferroptosis resistance in PDAC is therefore an essential prerequisite to establish more effective therapy strategies.

This review highlights the current understanding of the role of Nrf2, mitochondrial functioning, and ferroptosis in the development and progression of PDAC as one of the most fatal malignancies. Special emphasis is given on the underlying mechanisms by which Nrf2 and its signaling pathways (regulated antioxidant genes) are involved in mitochondrial function promote resistance to ferroptosis. This will be particularly discussed on pancreatic cancer and it’s still highly limited curability. We will further present an update on the most recently studied Nrf2 inhibitors, of both natural and synthetic origin, with potential therapeutic properties for pancreatic cancer.

## 2. Oxidative Stress and Nrf2

The most relevant ROS are hydrogen peroxide (H_2_O_2_) and the anionic superoxide radical (O_2_^−^) as well as the hydroxyl radical (OH^−^) that derives from H_2_O_2_ catalyzed by iron (Fe^2+^) during ferroptosis (see below). H_2_O_2_, through reversible oxidation of regulatory proteins (most of them containing reactive thiols), drives several biological processes including cell migration and cell growth, differentiation, or angiogenesis [28]. These actions are seen at intracellular H_2_O_2_ concentrations between 1 and 100 nM (or 100-fold higher extracellular H_2_O_2_) and are regarded as a homeostatic condition termed oxidative eustress [1]. If moderately elevated, e.g., during inflammation or due to metabolic alterations, H_2_O_2_ levels reach intracellular concentrations up to 1 µM (extracellular up to 100 µM), leading to numerous adaptive and compensatory events for the protection of the cell from ROS induced damage [1,28]. This includes DNA repair, cell cycle control, proteasome activation, unfolded protein response, metabolic alterations, and neutralization of excessive ROS. As this condition ensures the integrity and survival of cells, it can be regarded as adapted oxidative eustress. Important mediators of the adaptation to elevated H_2_O_2_ levels include transcription factors such as Forkhead box O (FOXO), tumor protein P53 (p53), hypoxia-inducible factor (HIF), Nuclear factor kappa-light-chain-enhancer of activated B cells (NF-κB), and Nrf2. If these adaptive events fail or if intracellular H_2_O_2_ level exceed 1 µM (or 100 µM extracellular), as seen upon extracellular insults like irradiation, exposure to anti-cancer drugs and certain chemicals or an aggravated inflammation, cell damage becomes irreversible finally leading to cell death. This condition of an insufficiently compensated insult through ROS is termed oxidative distress [1]. Notably, tumor cells—including PDAC cells—are very often more efficiently adapted to elevated levels of H_2_O_2_ and other ROS, thus being more protected from oxidative distress [13,14]. This resistance to oxidative distress is regarded as an essential mechanism underlying the phenotype plasticity of PDAC cells accounting for their aggressiveness and metastatic potential [29]. A prerequisite for the adaptation to ROS and therefore an important tumor promoting mechanism is the activation of Nrf2. This transcription factor is regarded as the master regulator of the cellular antioxidant system crucial for the activation of numerous cytoprotective genes as adaptive response to oxidative stress (OS) [30].

Nrf2 can be either activated via canonical and non-canonical mechanisms [31,32]. The canonical activation is dependent on the Keap1. Under basal non-stressed conditions, the expression of Nrf2 is kept at low level by its interaction with Keap1, which acts as a substrate carrier for the cullin 3 (Cul3)/RING-box protein 1 (RBX1) E3-ubiquitin ligase complex leading to Nrf2 ubiquitylation and proteasomal degradation. Under conditions of OS and electrophiles, modifications of free cysteine residues in the Keap1 protein as well as phosphorylation of Nrf2 prevent its ubiquitylation and proteasomal degradation [33]. Since Nrf2 remains bound to Keap1, the now saturated Keap1/Nrf2 complexes are not accessible for steady state de-novo synthesized Nrf2 that, accompanied by phosphorylation (e.g., at Ser-40), translocates into the nucleus where it binds to the ARE promoters and activates the transcription of various antioxidant as well as other target genes [4,5,6,7]. The most studied non-canonical mechanism of Nrf2 induction is through the nuclear pore glycoprotein (p62) protein, which is a receptor of the autophagy process. p62 plays an important role in removing defected proteins and mitochondria. It interacts with Keap1 via its KIR domain and competes with the ETGE motif of Nrf2 for Keap1 binding. The interplay between the Keap1 and p62 promotes the degradation of Keap1 and leads to the release of Nrf2 and subsequently to its nuclear translocation [7,31,32,33]. Likewise, non-electrophilic inducers of Nrf2 lead to its dissociation from Keap1 dimers [33]. Besides Keap1, F-box/WD repeat-containing protein 1A (β-TRCP) as another E3-ligase substrate carrier of Nrf2 has been identified and Nrf2 activation can occur through the glycogen synthase kinase-3 beta (GSK3β)/β-TRCP pathway [32].

### Impact of Nrf2 on Mitochondrial Function

In addition to the neutralization and scavenging of ROS in the cytoplasm, the antioxidant action of Nrf2 includes a wide range of effects on mitochondrial functions. Besides their role in providing energy through oxidative phosphorylation (OXPHOS), mitochondria are also responsible for other key functions of eukaryotic cells including multiple biosynthetic and regulatory functions. Moreover, mitochondria are an important source of ROS, largely produced in the electron transfer chain (ETC), and consequently subject of ROS controlling mechanisms [6] as an essential part of cellular homeostasis. This includes the regulation of programmed cell death through apoptosis and ferroptosis [34,35], as described below (Section 4). Given these crucial roles of mitochondria, besides energy metabolism, abnormal mitochondrial functions are the cause of various diseases including cancer [36].

**Figure 1:**
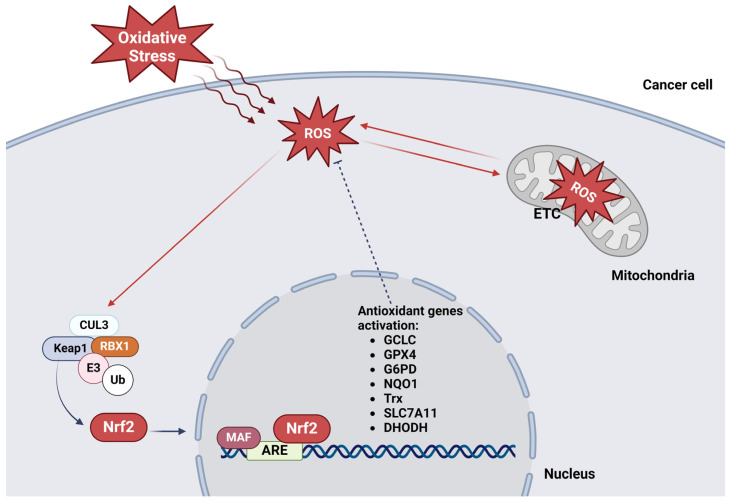
Nrf2 in the regulation of mitochondrial ROS (mROS). Oxidative stress induces mROS generation thereby activating Nrf2. After translocation into the nucleus, Nrf2 binds to the ARE in the promoter region of antioxidant genes. The elevated expression of these Nrf2 target genes contributes to mROS scavenging thereby counteracting the deleterious effects of ROS on mitochondrial function which are also part of ferroptosis initiation. For details, refer to the text in Section Impact of Nrf2 on Mitochondrial Function and Section 4 (The figure is generated in BioRender.com).

Many processes underlying the functions and activity of mitochondria are controlled by Nrf2 in different ways (Figure 1). It is well established that Nrf2-induced gene expression plays a fundamental role in mitochondrial processes governing their functional homeostasis and biology, energetics, fatty acid oxidation, or the production of adenosine triphosphate (ATP). This also affects respirational processes, as well as the dynamics of mitochondria [35]. Furthermore, it is known that Nrf2 encodes a battery of antioxidant genes (*Glutamate–cysteine ligase catalytic subunit (GCLC), NAD(P)H dehydrogenase [quinone] 1 (NQO1), heme oxygenase 1 gene (HMOX1), Thioredoxin (Trx), Glucose-6-Phosphat-Dehydrogenase (G6PD)*, etc.) that orchestrate the mitochondrial metabolism of iron or the production of nicotinamide adenine dinucleotide phosphate (NADPH) as well as other homeostatic processes in this organelle [37] (Figure 1).

Besides the above-mentioned functions, Nrf2 maintains redox homeostasis by controlling ROS accumulation through mitochondrial glutathione (mGSH) metabolism and NADPH synthesis [38]. For the recovery of the mGSH pool, which is exploited by enzymatic oxidation by GSH peroxidases to neutralize hydrogen peroxide (H_2_O_2_) and hydroperoxides, the formed glutathione disulfide (GSSG) is converted back to GSH by NADPH-dependent GSH-reductase controlled by Nrf2 [36,39,40]. Moreover, Nrf2 induces GSH de-novo synthesis [41] via its target gene *GCLC*. Accordingly, Nrf2 can be regarded as key regulator of the GSH-dependent antioxidant system. In addition, to neutralize ROS overproduction along with OS, Nrf2 exerts its antioxidant function by inducing expression of mitochondrial enzymes including thioredoxin 2 (TRX2), peroxiredoxin 3 (Prdx3), peroxiredoxin 5 (Prdx5), superoxide dismutase 2 (SOD2), etc. [42,43,44]. Activation of Nrf2 also impacts mitochondrial biogenesis through controlling the expression of NR1C3 (nuclear receptor subfamily 1, group C, member 3), which is the crucial transcriptional factor involved in lipid and glucose metabolism.

Through these mechanisms, mitochondria adapt to raised OS and prevent OS-induced functional and structural failure. Accordingly, impaired Nrf2 activation leads to damage of mitochondria, supporting the view that engagement of Nrf2 is the crucial protective process under the conditions of mitochondrial OS and oxidative lesion [45]. This requires sufficient signaling between mitochondria and the cytosolic Keap1-Nrf2 complex. Besides the direct export of mROS, changes in mitochondrial redox homeostasis contribute to secondary signals that promote activation of Nrf2 in the cytosol. Thus, complex formation between Keap1 and the mitochondrial enzyme PGAM family member 5 (PGAM5), an effector of necrosis caused by ROS [39], activates Nrf2 [45,46]. In another study, it was found that eriodictyol, a flavonoid isolated from plants, fruits, and vegetables, has a strong effect on cell viability, mitochondrial function, and ferroptosis by suppressing the Nrf2—heme oxygenase 1 (HO-1)—NQO1 regulatory axis in ovarian cancer cells [47]. Moreover, in vivo study demonstrated an inhibitory effect of the compound on tumor growth, mitochondrial function, and Nrf2 expression levels in mice [47]. This further confirms the significance of Nrf2 and its target genes in regulating mitochondrial functions. Accordingly, the execution of ferroptosis as another mitochondrial process (see Section 4) is under control of Nrf2, as well.

## 3. Deregulation of Nrf2 and Its Function in PDAC

While loss-of-function mutations in *KEAP1* preventing the control of Nrf2 stability have been reported to occur in different cancers frequently, e.g., in lung or esophageal cancer, these mutational events are less frequent in PDAC. Likewise, gain-of function mutations in Nrf2, affecting its Keap1 interaction and degradation [48], are rarely found in PDAC. Instead, epigenetic control of Keap1 and an increased expression level of competitive inhibitors of NRF2 binding to Keap1 also contribute to NRF2 activation in several tumor entities including pancreatic cancer [49]. Thus, aberrant hypermethylation of the *KEAP1* gene has been shown to be associated with poor prognosis of pancreatic cancer [3]. It was shown that the hemi-methylated DNA interacting protein ubiquitin-like, containing PHD and RING finger domains 1 (UHRF1) and the CpG island interacting protein Methyl-CpG Binding Domain Protein 1 (MBD1), promote the methylation of the *KEAP1* gene promoter causing deregulation of Nrf2 in PDAC cells [3,50]. Moreover, lncRNAs and microRNAs, playing an important role in the control of Nrf2 [51,52,53], have been identified in pancreatic carcinogenesis as well. The lncRNA SLC7A11-AS1 was recently shown to induce drug-resistance of PDAC cells through targeting the non-canonical GSK3β/β-TRCP pathway [54]. Another study revealed that the microRNAs miR-93 and miR-200a that target Nrf2 and Keap1 expression, respectively, differentially affect PDAC progression and patient survival [55]. 

Besides epigenetic effects underlying deregulated Nrf2 activation, alternative Nrf2-activation pathways have been reported in pancreatic cancer as well. Recent studies demonstrated the non-canonical activation of Nrf2 through p62 [56] or through protein kinase R (PKR)-like endoplasmic reticulum kinase (PERK)-dependent phosphorylation of Nrf2 and the prevention of its interaction with Keap1 under hypoxic conditions [57]. Other studies reported that overexpression of partner and localizer of BRCA2 (PALB2) [58], ataxia-telangiectasia group D-associated (ATDC) gene products [59], atypical PKCι [60], and carnitine palmitoyl transferase type IB (CPT-IB) [61] prevent the interaction of KEAP1 and Nrf2, accounting for its persistent activation in PDAC. It was also demonstrated that overexpressed ubiquitin-specific protease 8 (USP8) leads to deubiquitylation of Nrf2 and thereby to its retrieval from proteasomal degradation [62]. Moreover, in an indirect fashion, the amplified kRas/ERK [63,64,65] and glucose-regulated protein 78/unfolded protein response [66] pathways, overexpression of the glycolysis enzyme aldolase-A [67], or an elevated activation of signal transducer and activator of transcription 3 (STAT3) [68] have all been identified to upregulate Nrf2 activity in PDAC cells. Finally, the profound tumor stroma in PDAC [69], consisting of cancer associated fibroblasts, largely myo-(myCAFs), and inflammatory fibroblasts (iCAFs) [70], myeloid-derived suppressor cells (MDSCs), macrophages, and T-cells, is a source of persistent OS to which PDAC cells need to adapt through enhanced Nrf2 activity. This stroma derived OS is due to the release of ROS (mainly H_2_O_2_ and NO) by MDSCs and macrophages [49] as well as of ROS-inducing cytokines such as IL-6 and TGFβ secreted by iCAFs and myCAFs, respectively [71,72]. Moreover, the metabolite competition by the highly glycolytic myCAFs consuming large amounts of glucose and releasing pyruvate and lactate [73] increases OS in the PDAC cells. Recently, it was further demonstrated that the prolyl isomerase PIN1, which is an important modulator of the ECM composition and its stiffness in PDAC [74], induces Nrf2 through enhancing cMyc induced Nrf2 expression [75].

According to studies published so far, Nrf2 has a dual role in pancreatic cancer. It exerts tumor suppressive effects at the stage prior to tumor initiation [14,76], mainly depending on the protection of pancreatic duct cells from ROS-induced DNA damage and thereby mutational events. This condition can manifest during pancreatic injuries caused by high-fat diet, smoking, and alcohol consumption, leading to an acute pancreatitis accompanied by a forced ROS production [77]. However, during the early onset of carcinogenesis and later at advanced stages, Nrf2-induced processes favor malignant transformation and support tumor growth, as evidenced in genetic mouse models of PDAC [6,10]. This includes profound supportive effects of Nrf2 on kRas dependent initiation of pancreatic carcinogenesis [13,78,79], seen as early as the premalignant stages [80], and further on in cooperation with mutated p53 [81] and through differential splicing of activating transcription factor 3 (ATF3) [82]. Accordingly, Nrf2 has been shown to be fundamentally active in pancreatic cancer and associated with a poor prognosis [13,83,84]. Thus, Nrf2 provides premalignant and malignant pancreatic duct cells with defense mechanisms against the deleterious effects of ROS, thereby overriding growth arrest and preventing cell death [13,14]. This includes not only antioxidant and ROS neutralizing enzymes but also other regulatory proteins e.g., proteolytic homeostasis through the 26S-proteasome [85]. In addition, Nrf2 activity, in concert with HIF1 and cMyc, induces metabolic alterations through an increased expression of glycolytic enzymes and enzymes involved in the PPP [67]. Another important action of Nrf2 is the induction of the epithelial mesenchymal transition through its cooperation with STAT3 [68] or TGFß1 in a JNK-dependent fashion and altered expression level of epithelial-to-mesenchymal transition (EMT) related genes, like E-cadherin, Vimentin, Slug, and Snail [86]. Moreover, Nrf2 favors EMT in premalignant pancreatic duct cells [80,86]. More recently, it was shown that through its effect on lactate uptake and metabolism in PDAC cells, Nrf2 provides greater resilience to glucose and glutamine scarcity [87,88] accompanied by an increased stemness potential and greater chemoresistance [87,89,90].

Given these cellular alterations, Nrf2 essentially adds to the plasticity of PDAC cells accounting for their greater migratory and metastatic potential as well as their resistance against the established anti-cancer drugs gemcitabine and 5-FU [85,89,91,92]. An important mechanism of the killing effect of these drugs on tumor cells is an increased ROS production along with the generation of OH-radicals that drive programmed cell death through ferroptosis [54,61,93]. Evidence has accumulated that Nrf2 essentially contributes to the resistance of PDAC cells to ferroptosis and thereby to the failure of drug induced cell killing. Therefore, overcoming Nrf2-dependent ferroptosis resistance is an attractive approach to greatly improve the outcome of the above drug treatments [27].

## 4. Molecular Mechanisms Underlying Ferroptosis

In the past decade, significant attention has been paid to regulated cell death, named ferroptosis, that is dependent on iron and characterized by the accumulation of lipid oxidation products (LOPs) on the cell membrane [16,25]. In physiological conditions, ferroptosis promotes tumor suppressor activation, thus preventing tumor progression [94,95]. Nevertheless, in advanced stages of malignant entities, cancerous cells develop mechanisms of ferroptosis resistance contributing to their accelerated proliferation, metastasis formation, and chemoresistance [94,96,97]. Due to high demands on DNA replication and proliferation rates, pancreatic cancer cells are highly dependent on intracellular iron [98]. Iron oxidation and reduction reactions induce production of ROS, thus allowing tumor cells to proliferate [99]. This type of cell death is morphologically characterized by a decrease in mitochondrial volume, increase in membrane density, reduction, or absence of the mitochondrial crista and rupture of the outer membrane. Biochemically, GSH depletion, glutathione peroxidase (GPX4) inactivation, accumulation of iron ions, and LOPs are observed in cells undergoing ferroptosis [100].

**Figure 2:**
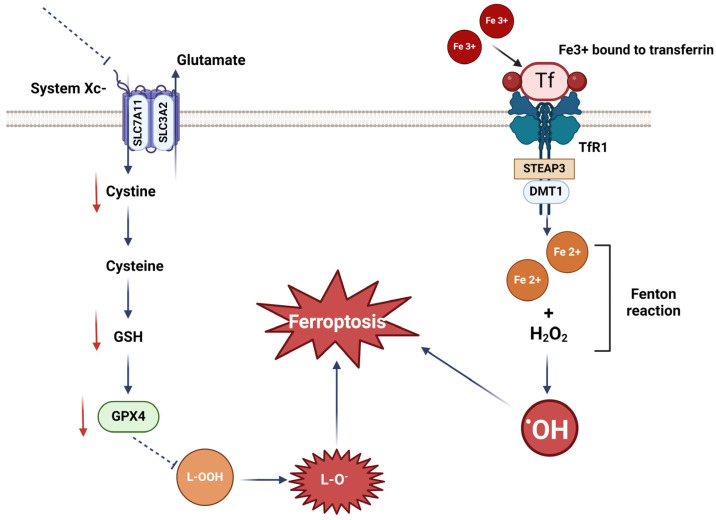
Molecular mechanisms of ferroptosis initiation. The two main pathways in ferroptosis initiation include the impairment of the cellular GSH antioxidant system compromising GPX4 dependent protection from lipid-peroxidation and the altered iron metabolism leading to Fenton reaction. For details, refer to the text in Section 4. (The figure is generated in BioRender.com).

Ferroptosis is initiated by insufficiency of the GSH-dependent antioxidant system, which, in turn, leads to iron-promoted lipid peroxidation process, ROS overaccumulation, and, as a result, to cell death [101]. It can be activated by altered iron metabolism in a cell. Iron metabolism consists of iron import, storage, and export. Upon binding to transferrin (Tf), transferrin receptor 1 (Tfr1) transports ferric iron (Fe^3+^) to the endosome. Then, Fe^3+^ is reduced to ferrous iron (Fe^2+^), which is collected in a labile cytoplasmic iron pool through the divalent metal transporter (DMT1). At the final step, the excess of iron is released by iron regulated transporter 1 (IREG1) [102,103]. During ferroptosis, Fe^3+^ and OH^−^ are generated by the Fenton reaction when Fe^2+^ reacts with H_2_O_2_, thereby having a deleterious impact on the cell structures and further leading to cell death [30] (Figure 2). 

Except iron-dependent ROS aggregation, there are other causes of ferroptosis induction, such as autophagy, altered metabolism of lipids, amino acids, as well as changes in the tumor microenvironment [104,105]. Androgen Receptor Activator (ARA70), also known as nuclear receptor coactivator 4 (NCOA4), contributes to the release of Fe^2+^ by mediating ferritinophagy, the process of autophagic degradation of Ferritin (Ft). Under physiological state, ferritinophagy is tightly controlled, sustaining the balance of iron in cells. Nevertheless, overactivation of this process contributes to the excessive accumulation of iron in cells, leading to ROS overproduction and further to ferroptosis initiation [105,106]. Overexpression of ARA70 increases free iron levels in cells, which further leads to ferroptotic cell death. Likewise, activation of autophagy proteins 5 and 7 (ATG5 and ATG7, respectively) in the ATG5/7-ARA70 regulatory pathway suppresses the expression of the ferritin heavy chain (FTH1), thus compromising Ft function in PDAC cells and thereby contributing to the elevation of intracellular Fe^2+^ and lipid ROS that leads to ferroptosis initiation [107].

Another regulator of ferroptotic cell death is the anionic amino acid transport system Xc-, which consists of solute carrier family 3 member 2 (SLC3A2) and solute carrier family 7 member 11 (SLC7A11). These proteins import extracellular cystine and export intracellular l-glutamate across the cellular plasma membrane. Cystine is reduced to cysteine for GSH synthesis and is thereby essential for the control of lipid peroxidation by GPX4. The mechanism of action of GPX4 is to catalyze the reduction of deleterious lipid peroxides (L-OOH) at the expense of reduced GSH and yielding lipid alcohols (L-OH), thereby preventing ferroptosis. Consequently, GPX4 inactivation leads to the formation of L-OOH aggregates that are converted by iron into toxic lipid radicals (alkoxy radical (L-O)) leading to the ferroptotic cell death [108] (Figure 2). Likewise, inhibition of the system Xc-causes ferroptosis due to a decrease of intracellular cysteine [109] and thereby GSH levels. This accounts for a reduced GPX4 driven neutralization of lipid peroxides and consequently leads to the initiation of ferroptosis. Through this mechanism, Xc- inhibition diminishes proliferative rates and survival of PDAC cells [95,110]. Finally, ferroptosis can be also initiated by the tumor microenvironment (TME) in PDAC. Wang et al. showed that CD8+ T cells in the TME inhibit the expression of SLC7A11 and SLC3A2 in cancer cells through secretion of gamma interferon (IFN-γ). This restricts cystine import into the cancer cells, thereby inducing lipid peroxidation and, as a consequence, ferroptotic cell death [111].

Some studies revealed that increased GPX4 expression is associated with better prognosis in PDAC patients [112]. There is also a correlation between GPX4 expression and elevated survival rates of pancreatic cancer patients, indicating that it can be a valuable prognostic marker [102]. In addition, it was observed that mitochondrial Lon peptidase 1 (LONP1) (an enzyme regulating mitochondrial function) contributes to tumor suppression by decreasing GPX4 expression and thereby affecting neutralization of L-OOH levels in PDAC cells. Moreover, LONP1 interferes with the Nrf2-Keap1 axis, thus negatively influencing cancer cell growth [95,113].

### 4.1. Role of Phospholipids in Ferroptosis

Fatty acids shape membrane phospholipids and are important for lipid peroxidation. Phospholipids interact with oxygen and neighboring lipids to produce phospholipid hydroperoxides (PL-OOH). The lipid peroxidation process requires phospholipid radicals, which are products of interaction between Fe^2+^ and PL-OOH [114]. Products of PL-OOH decomposition lead to the rupture of the cell membrane. Extensive lipid peroxidation, in turn, contributes to cell death. LOPs promote damage of cellular structures by their decomposition into ROS and by alterations in the physical structure of membranes. These events contribute to the release of detrimental substances, thereby compromising the cellular metabolism. 

### 4.2. Role of Mitochondrial Iron Metabolism in Ferroptosis

Iron is transported from the cytosol to mitochondria by the membrane transferrin receptor (TfR). The vast amount of cellular iron is bound by Ft, while the remaining amount is transferred to the mitochondrion through the iron transporter: mitoferrin-1 (Mfrn1) in erythroblasts and by Mfrn2 in non-erythroid cells [115]. Mitochondrial iron storage and its homeostasis are regulated by the mitochondrial ferritin (FtMt) [116]. It is mainly metabolized in the mitochondrial matrix. FtMt serves for the generation of heme and Fe-S clusters as cofactors of various enzymes implicated in DNA replication and repair, redox reactions, and other essential cellular processes [116,117]. FtMt executes cellular defense functions against mROS accumulation, and its malfunction results in iron and ROS overload, further leading to ferroptosis [118,119]. Excess iron results in mitochondrial dysfunction, which manifests in ROS accumulation, inhibition of mitochondrial respiration, and a decrease in the mitochondrial membrane potential [120]. Impairment of the iron ion transport and homeostasis leads to its accumulation, giving rise to damage of nucleic acids, proteins, and lipids, causing OS, and further ferroptosis [121].

### 4.3. Role of Mitochondrial GPX4 and DHODH Systems in Ferroptosis

The two main defense mechanisms against iron dependent cell death rely on dihydroorotate dehydrogenase (DHODH) and glutathione peroxidase 4 (GPX4) [122], which are localized on the outer surface of the IMM, and on the mitochondrion and cytoplasm, respectively. GPX4 acts by inhibition of the lipid peroxidation process via degradation of small molecular peroxides and composite LOPs, thus antagonizing ferroptosis [123]. Under OS, GPX4 ensures the generation of mitochondrial ATP and supports the mitochondrial membrane potential [15]. GPX4 expression in mitochondria diminishes peroxidation of lipids, thus antagonizing ferroptosis (see above). Particularly, GPX4 preserves cellular structures from the damage by oxidation of low-density plasma lipoprotein particles and Chol-OOH (cholesterol hydroperoxides) that promote iron dependent cell death [115]. DHODH is involved in the electron transfer in the ETC. Moreover, DHODH converts CoQ to CoQH2 and the DHODH-CoQh2 system shuts down the peroxidation of lipids in mitochondria and thus ferroptosis [122,124,125]. 

In a recent study, Shiqi Wua et al. have detected that treatment of tumorous cells with sn-Glycerol 3-phosphate (G3P) impairs iron cell death induced by GPX4 inhibitors in a Glycerol-3-phosphate dehydrogenase 2 (GPD2)-dependent manner. Moreover, they have shown that GPD2 depletion sensitizes tumor cells to mitochondrial lipid peroxidation and ferroptosis induced by GPX4 suppression. Finally, it was demonstrated by an in vivo study that combined GPX4 and GPD2 depletion inhibits cancer cell proliferation via induction of iron cell death. Altogether, these results suggest that GPD2 plays a particular role in mitochondrial ferroptosis via ubiquinol production [126].

### 4.4. Role of mROS in Ferroptosis

Another example of the role of mitochondria in ferroptosis is the generation of large amounts of ROS by this organelle, leading to lipid peroxidation and to iron dependent cell death. ROS are produced mainly by NADPH oxidases. Under physiological conditions, ROS regulate cellular homeostasis and signaling (see above). However, ROS overload may contribute to metabolic dysfunction [127]. Complex I and Complex III of the ETC are the primary source of ROS generation. Superoxide radicals are generated through complex I and then emitted into the mitochondrial matrix. When the mitochondrial complexes I and III are suppressed, ROS are produced by complex II through the electron transport from succinate or from reduced ubiquinone [116].

The antioxidant system consists of the GSH redox system (GPX), Glutathiondisulfid-Reductase (GSR), and peroxiredoxins (Prxs)), Trx, thioltransferase, or glutaredoxin system. Normally, hydrogen peroxide is converted by the GSH redox system to O_2_ and H_2_O [128]. Dysfunctionality of the cellular antioxidant system therefore contributes to an overload of cells with ROS and thereby leads to peroxidation of lipids on the cell membrane and, as a consequence, to ferroptosis. Thus, the excessive mROS accumulation contributes to the peroxidation of lipids on the cell membrane and consequently to the iron cell death [116].

### 4.5. Role of Mitochondrial Citric acid Cycle in Ferroptosis

The non-essential alpha-amino acid glutamine is one source of carbon for the mitochondrial citric acid cycle. It is converted by the mitochondrial enzyme glutaminase (GLS) into glutamate and then by Glutamic-oxaloacetic transaminase (GOT1) into α-Ketoglutaric acid (α-KG), which serves as a fuel for the citric acid cycle [129]. Intriguingly, suppression of the citric acid cycle may contribute to ferroptosis [15,130,131] it is assumed that the citric acid cycle regulates iron cell death via sustaining mitochondrial ETC, which facilitates generation of ROS [132]. Accordingly, resistance to iron cell death is caused by loss-of-function mutations in the citric acid cycle enzyme fumarase [133]. These observations suggest that the mitochondrial citric acid cycle is involved in the regulation of ferroptosis. Finally, LONP1 [113] and BH3 interacting-domain death agonist (BID) are modulators of iron cell death [134], and a number of ferroptosis proteins including ASCL4 and P53 show partial mitochondrial localization [115].

### 4.6. Role of Mitochondrial NADPH in Ferroptosis

It is well known that mitochondria are the main organelles for the production and metabolism of the important electron donor NADPH [135], which is generated by isocitrate dehydrogenase 1 (IDH1) and malic enzyme (ME) during the citric acid cycle [115]. NADPH has a significant impact on ferroptosis [136] as it, in combination with Ferroptosis suppressor protein 1 (FSP1), contributes to the neutralization of lipophilic radicals, thereby reducing the accumulation of LOPs and suppressing iron cell death [137,138]. Notably, the mitochondrial form of isocitrate dehydrogenase 2 (IDH2), which is associated with greater cancer malignancy, catalyzes the oxidative decarboxylation of isocitrate into alpha-ketoglutarate by reducing NADP+ to NADPH, and silencing of IDH2 leads to a significant enhancement in the susceptibility of cancer cells to ferroptosis [139].

### 4.7. Role of Mitochondrial MCU in Ferroptosis

Additional evidence for the correlation between mitochondria and ferroptosis, as well as their connection with the complex actions of Nrf2, came from a recent study by Wang et al. [140]. They demonstrated that overexpression of the mitochondrial calcium uniporter (MCU) in PDAC accounts for an enhanced mROS generation due to increased mCa2+ level that promotes the citric acid cycle and OXPHOS. On the one hand, this condition supports ferroptosis through upregulated lipid peroxidation, but on the other hand it induces the Keap1-Nrf2 pathway, depending on cystine availability. Notably, this mROS induced Keap1-Nrf2 response in MCU overexpressing PDAC cells not only prevents ferroptosis but also results in greater metastasis formation. In accordance with the cystine dependency of the Nrf2 activation, ferroptosis by MCU-triggered mROS formation is enhanced and PDAC metastasis is diminished if cystine is absent or if SLC7A11 is inhibited [140]. 

**Figure 3:**
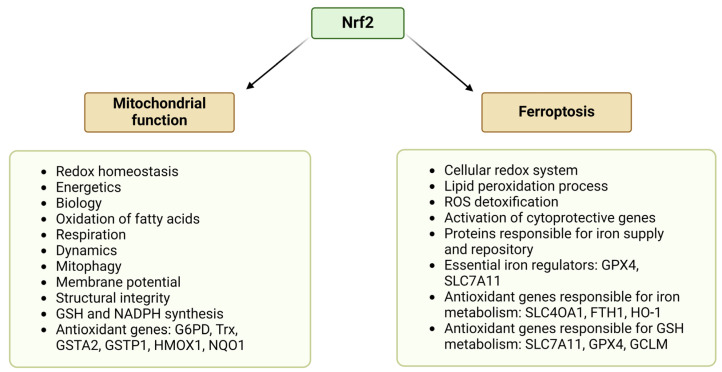
Role of Nrf2 in the regulation of mitochondrial functions and ferroptosis. Many processes controlling mitochondria and ferroptosis are orchestrated by Nrf2. For details, refer to the text in Section 2 and Section 5. (The figure is generated in BioRender.com).

Thus, mitochondria, in their function, are linked at several levels to the regulated cell death through ferroptosis. Given its pivotal and highly complex role in the regulation of mitochondrial activity and function, Nrf2 greatly impacts ferroptosis as well (Figure 3). This depends on its orchestration of the mitochondrial redox and iron homeostasis. Accordingly, interfering with Nrf2-dependent pathways controlling both mitochondrial processes and ferroptosis would enhance the ferroptotic death of PDAC cells.

## 5. Role of Nrf2 in the Control of Ferroptosis and Drug Resistance in Pancreatic Cancer

During recent years, a great number of experimental approaches emerged to induce ferroptosis as a measure to suppress PDAC growth [54,109,141]. Several compounds, such as thiostrepton [142], verteporfin [143], artesunate [144], or ruscogenin [145], have been described to efficiently kill PDAC cells through ferroptosis at different stages of the ferroptotic death program [108]. Notably, artesunate and ruscogenin have been reported to interfere with Nrf2 as well [146,147], thus indicating that these actions either cooperate to execute ferroptosis or that they may counteract depending on the status of Nrf2 activity. This, again, points to the need to unveil the mechanism behind the control of ferroptosis in PDAC by this transcription factor.

Nrf2 controls a variety of proteins responsible for iron supply, repository, and metabolism [148]. As many Nrf2 target genes are responsible for iron homeostasis and carrying out a protective role against ferroptosis, Nrf2 is involved in regulating peroxidation of lipids and iron cell death [149]. These genes include *GPX*, *SLC7A11*, and *HO-1* [16] (Figure 3). Nrf2-induced HO-1 is controlled by the Keap1/Nrf2/HO-1 regulatory axis and has protective potential against cytotoxicity of different OS and inflammation. Moreover, it has been revealed that Keap1-Nrf2-HO-1 regulatory pathway also controls the EMT process, and its activation depends on ferritinophagy-mediated ROS production [150].

Dodson et al. discovered that Nrf2 has a crucial role in peroxidation of lipids and ferroptosis. This is evidenced by the fact that SLC7A11 and GPX4, essential regulators of ferroptosis, are controlled by Nrf2 [149]. Silencing of the Nrf2 regulator Keap1 contributed to iron induced decrease in expression of GPX4 and SLC7A11 as well as increase in expression of Acyl-CoA Synthetase Long Chain Family Member 4 (ACSL4) proteins, accentuating the significance of the role of Nrf2 in ferroptosis. Their data indicate that iron regulates gpx4 and slc7a11 by the Nrf2-ARE signaling pathway. Therefore, SLC7A11 and GPX4 are implicated in the Nrf2-mediated defense against iron dependent cell death by direct binding of Nrf2 to gpx4 and slc7a11 promoter regions inducing their transcription and thus controlling ferroptosis [151]. In a study on pancreatic cancer cells, Liu X et al. revealed that an increased expression of the *NFE2L2* gene, encoding the Nrf2 protein, substantially reduces Fe^2+^ ion levels and lipid peroxidation, and leads to a decrease in GSH level after wogonin treatment. Conversely, the knockdown of *NFE2L2* dramatically increased these processes upon wogonin treatment. These results confirmed that the level of Nrf2 expression correlates with the susceptibility of cells to ferroptosis; an elevation of Nrf2 expression suppresses this type of regulated cell death. This suppression is due to upregulated GSH levels by increasing the expression of GPX4 and SLC7A11 by Nrf2 [90].

More recently, the role of Nrf2 in ferroptosis resistance of PDAC cells was also studied after CPT-IB knockdown [61]. The authors revealed that protein levels of Nrf2 and its target gene *HO-1* were reduced after CPT1B silencing, an effect depending on the presence of Keap1. This observation confirmed that CPT1B regulates Nrf2 via Keap1. Moreover, the authors showed that the expression level of GPX4 and the ferroptosis promoting gene *long-chain-fatty-acid-CoA ligase 4 (ACSL4)* were decreased and increased, respectively. Thereby, and depending on the Keap1-Nrf2 pathway, the knockdown of CPT1B reduced GSH and raised ROS and LOPs levels, leading to enhanced ferroptosis [61]. 

Tao et al. investigated Nrf2 and its target genes expression, including GPX4, ferritin heavy chain (FTH1), and Heat shock protein 90 (HSP90), in the pancreatic cell line MiaPaCa2. They have revealed that expression of these genes was substantially downregulated after brusatol treatment, confirming the effectiveness of this treatment on Nrf2 downregulation. Moreover, they discovered that ferroptosis was induced by downregulation of FTH1 and GPX4, thereby enhancing the anticancer properties of brusatol [152]. Another study indicated that inhibition of the Nrf2/HO-1 pathway stimulated ferroptosis upon treatment with cetuximab in KRAS mutant colorectal cancer [153]. Kuang et al. demonstrated that Nrf2-regulated microsomal glutathione S-transferase 1 (MGST1) expression contributes to ferroptosis inhibition in PDAC cells. Silencing of MGST1 diminished the resistance of PDAC cells to ferroptosis, while the re-expression of MGST1 restored the resistance of Nrf2-knockdown PDAC cells to ferroptosis [154]. 

Guo et al. demonstrated that the exosomal release of GOT1 inhibits ferroptosis and promotes growth of PDAC cells. This effect relies on an increased expression of the C-C chemokine receptor type 2 (CCR2), which activates the Nrf2/HO1 signaling pathway [155]. Moreover, Liu et al. demonstrated increased levels of ferrous iron promoted by the 5′ AMP-activated protein kinase (AMPK)/NRF2/HMOX1 regulatory axis after treatment of cells with vitamin C and the ferroptosis inducer erastin. Nrf2, as the crucial activator of HMOX1, can be also engaged in iron homeostasis via the regulation of FTH1 and ferroportin-1 (IREG1). It is recruited for autophagic degradation and iron dissociation by nuclear receptor co-activator 4 (NCOA4), thus promoting iron dependent cell death. In addition, stimulating AMPK may cause nuclear conglomeration of Nrf2 and exert a positive impact on the Nrf2/HMOX1 signaling pathway [156].

**Figure 4:**
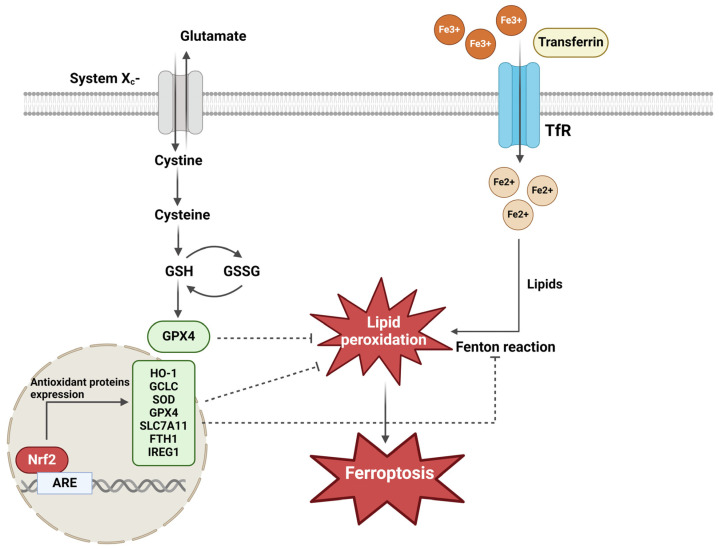
Regulation of the ferroptotic pathway by Nrf2. Ferroptotic cell death is prevented by the Nrf2 pathway through a battery of antioxidant proteins supporting the GSH-antioxidant system and inhibiting lipid-peroxidation (HO-1, Glutamate—cysteine ligase catalytic subunit (GCLC), GPX4, SLC7A11, DHODH). In addition, Nrf2 also prevents iron overload of cells by increasing ferritin (FTH1) and IREG1 expression. For details, refer to the text in Section 5. (The figure is generated in BioRender.com).

This indicates the importance of Nrf2 and its numerous downstream genes either in suppressing or promoting ferroptosis in different cancer cell lines, including pancreatic cancer (Figure 4).

### Nrf2 and Resistance to Gemcitabine Induced Ferroptosis in PDAC

Gemcitabine is one of the first-line chemotherapeutic drugs used for the treatment of pancreatic cancer patients. The mechanism of its action is to induce ROS aggregation [92,157] and thereby the OS pathway promoting the iron dependent cell death. As described above, cancer cells develop strong antioxidant defense systems due to several antioxidant enzymes, which are regulated by Nrf2 [158]. It is noteworthy that the level of Nrf2 expression is associated with the sensitivity of cancer cells to ferroptosis. Elevated Nrf2 expression prevents ferroptosis, while its downregulated expression enhances the sensitivity of PDAC cells to this type of cell death [16,159,160]. Thus, Nrf2 essentially controls the expression of key proteins controlling the iron dependent cell death. Nrf2 induces transcription of target genes like *G6PD*, *SLC7A11*, and *FTH1* [149] that are essentially involved in the regulatory process of lipid peroxidation and iron metabolism. In line with this, a study on pancreatic cancer cells indicated that plasma-treated, water-derived antioxidants target the Nrf2-HMOX1-GPX4 signaling axis, thereby sensitizing cells to ferroptosis [161]. This suggests that inhibitors of Nrf2 and its target genes may have a great potential in promoting the iron dependent cell death as well as in overcoming gemcitabine resistance in pancreatic cancer cells.

Given the important role of the Xc-—GPX4 axis in the regulation of ferroptosis, functional suppression of the two genes may help to overcome the chemo- and radio-resistance of cancer cells. Previous studies revealed that activation of heat shock 70 kDa protein 5 (HSPA5)-GPX4 signaling contributes to the resistance of PDAC cells to gemcitabine. Accordingly, the suppression of the HSPA5-GPX4 pathway increases the sensitivity of PDAC to gemcitabine by enhancing ferroptosis [105,162]. Furthermore, suppression of the Xc-transport system promotes the cytotoxicity of gemcitabine in PDAC cells [133]. Another study demonstrated, in a genetic mouse model (KPC mice), that pancreatic cancer cells require cystine to escape ferroptosis and to survive suppression of SLC7A11 [138]. It was also suggested that gemcitabine activates NOX-induced ROS aggregation via NF-κB activation. This, in turn, contributes to the activation of Nrf2 and the elevation of cellular GSH levels contributing to resistance of PDAC cells. Thus, Nrf2 suppression in combination with stimulation of ferroptosis may have a considerable effect in overcoming gemcitabine resistance in PDAC [54]. It was demonstrated that combined treatment with chemotherapeutics and ferroptosis inducers improves the efficacy of chemotherapy. In another study, it was found that expression of SLC7A11 and SLC3A2 in parental pancreatic cancer cell lines correlates with gemcitabine resistance of PDAC patients [163]. Moreover, expression of the Xc-components dramatically increased in PDAC cell lines treated with gemcitabine. Thus, targeting the Xc-system may be highly efficient to improve the therapeutic outcome of gemcitabine-resistant PDAC.

Combination therapy for activation of ferroptosis in pancreatic cancer cells may have significant potential in becoming a promising research area by reversing the gemcitabine resistance, thus providing a strong ground for developing novel therapeutics to combat PDAC.

## 6. Nrf2 Inhibitors

Given the fact that Nrf2 activity is elevated at an advanced stage of PDAC, and having recognized the protection from ferroptosis by Nrf2 as a key mechanism in chemoresistance, strategies to block Nrf2-mediated cytoprotective pathways are an attractive option for anticancer therapy, especially if combined with conventional treatments. Nowadays, numerous substances of both natural and synthetic origin with Nrf2 inhibitory properties exist [164,165]. These compounds, with suppressive impact on Nrf2, have great potential anticancer effects, which have already been tested for different types of tumors, including PDAC, and many of them are currently in clinical testing. 

However, natural compounds often exert other cellular activities beside direct inhibition of Nrf2, e.g., by modulating NF-kB or STAT3 dependent signaling pathways or even by indirectly inducing the Nrf2-pathway. Thus, the applicability and safety of these compounds is rather limited when considered for Nrf2-targeted treatment concepts. Thus, synthetic drugs with greater selectivity in Nrf2 inhibition are needed. Much effort has been expended in recent years to design and develop such compounds. In this section, we highlight the most recent studied natural and synthetic compounds with Nrf2 inhibitory activity that exhibit promising therapeutic potential in different types of cancer, including PDAC, and that were shown to target Nrf2-induced resistance to ferroptosis. This is summed up in Table 1 and Table 2 (compounds tested with PDAC are highlighted in bold).

Among more than 20 natural compounds, brusatol was the most intensively studied, giving promising results with respect to its use as an Nrf2 inhibitor in anticancer treatment [166,167]. Notably, brusatol inhibits Nrf2-induced resistance to ferroptosis in lung, pancreatic, and esophageal cancers [61,168,169,170], adding to the broad range of previously reported effects through its inhibition of Nrf2. Likewise, wogonin was shown to block Nrf2-induced resistance to ferroptosis [90]. Trigonelline, as another natural Nrf2 inhibitor, attenuates chemoresistance of PDAC cells [85,171] and has been also shown to efficiently suppress Nrf2-induced ferroptosis resistance in head-neck, breast, and lung cancer cells [85,168,169,170,171,172,173,174]. Digoxin and brucein-D were shown to inhibit Nrf2-dependent gemcitabine resistance of PDAC cells [173,175]. Several other natural compounds, including triptolide, baicalin, luteolin, and erianin, were reported to abolish Nrf2-induced resistance to ferroptosis in various cancer types [90,176,177,178,179,180,181,182] but have not been studied in PDAC yet. 

Among the synthetic Nrf2 inhibitors, PIK-75 and tranylcypromine have been shown to block Nrf2-induced resistance to gemcitabine in PDAC cells [183,184]. Other synthetic compounds, including pizotifen malate and ML385 [185,186], have been shown to induce ferroptosis through Nrf2 inhibition in esophageal [168] and cholangial cancers [187] but have been not studied in PDAC yet. 

**Table 1:** Natural Nrf2 inhibitors. Compounds tested with PDAC are highlighted in bold.

#	Compound	Mechanism of Action	Type of Tumor /Cell Line	References
1.	Gossypol	Decreases NRF2 protein stability, suppresses the NRF2/ARE pathway, decreases the expression of Nrf2 downstream genes	Human head and neck squamous cell carcinoma (HNSCC) (TW 2.6, SCC-15, HSC-3), Human lung carcinoma (NCIeH460)	[188]
2.	Baicalin	Suppresses expression of Nrf2 downstream genes *GPX4* and *xCT*, via inducing ubiquitin degradation thus promoting ferroptosis	Human osteosarcoma cell lines (MG63, 143B)	[179]
3.	L-selenocystine	Inhibits Nrf2 expression and interferes with p62/Keap1/Nrf2 signaling pathway, thus leading to overproduction of intracellular ROS level	Human colorectal cancer (WiDr, C2Bbe1)	[189]
4.	Camptothecin	Suppresses SCL7A11 and Nrf2	Human hepatocellular carcinoma (HepG2, Huh7)	[190]
5.	Tangeretin	Suppresses the Nrf2 pathway, thus enhancing ROS generation	Human non-small cell lung cancer (NSCLC) (NCI-H1819, A549, NCI-H1975, HCC827)	[191]
6.	Elaiophylin	Inhibits mitophagy, increases OS. Suppresses deacetylation of Nrf2 in a SIRT1-dependent manner, thus causing elevation of non-functional Nrf2 in the cytoplasm	Human lung adenocarcinoma (A549, H1975, Calu-3)	[192]
7.	Erianin	Inhibits the Nrf2 signaling pathway, thus inducing ferroptosis and cell cycle arrest	Human bladder cancer (RT4, KU-19–19)	[182]
8.	Kaempferol	Inhibits the Nrf2 luciferase activity, further suppressing Nrf2 and its downstream targets. Suppresses the Nrf2 pathway via degrading Nrf2 mRNA	Human lung adenocarcinoma (A549 and NCIH460)	[193]
9.	S-3′-hydroxy- 7′, 2′, 4′-trimethoxyisoxane	Induces ferroptosis via Nrf2/HO-1 signaling pathway. Disrupts iron homeostasis, elevates Fe^2+^ counts, decreases the GSH level, downregulates GPX4, promotes lipid peroxides accumulation.	Human gastric cancer (SGC-7901), Human lung adenocarcinoma (A549 and H460), Human colorectal cancer (SW480), Human liver cancer (BEL-7402), Human breast cancer (MCF-7), Human cervical cancer (Hela), Human lung epithelial carcinoma (HBE)	[194]
10.	**Brucein D**	Downregulates the Nrf2 pathway via ubiquitin–proteasome-dependent Nrf2 degradation	Human pancreatic ductal adenocarcinoma (PANC-1, Capan-2, Miapaca-2)	[175]
11.	**Wogonin**	Inhibits Nrf2/GPX4 regulatory pathway, reduces GSH levels, induces ferroptosis.	Human pancreatic ductal adenocarcinoma (AsPC-1, PANC-1)	[90]
12.	Tetrahydroanthraquinone	Downregulates Nrf2 and its targeted antioxidant genes.	Human breast adenocarcinoma (MCF-7)	[195]
13.	Ursolic acid	Inhibits EGF-induced EGFR phosphorylation and Nrf2 phosphorylation.	Human breast cancer (MDA-MB-231)	[196]
14.	Cardamonin	Inhibits the Nrf2-dependent ROS scavenging system increase in intracellular ROS levels.	Human breast cancer (MDA-MB-231)	[197]
15.	Trabectedin	Promotes ferroptosis by regulating the Keap1/Nrf2/GPX4 pathway.	Human NSCLC (A549, H460, PC-9, H1299)	[198]
16.	Ginkgetin	Inactivates the Nrf2/HO-1 axis, thus enhancing ROS production and disrupting redox homeostasis.	Human NSCLC (A549, NCI-H460, SPC-A-1)	[199]
17.	**Brusatol**	Suppresses Nrf2 and its target genes, increases ROS generation.	Human pancreatic ductal adenocarcinoma (Panc1, MiaPaCa-2, Colo357)	[84,166,167]
18.	**Trigonelline**	Inhibits nuclear translocation of Nrf2 thereby blocking expression of its proteasomal target genes. Reversal of apoptosis resistance	Human pancreatic ductal adenocarcinoma (Panc1, MiaPaCa-2, Colo357), Human head & neck cancer (AMC-HN2–11, SNU- -1041, −1066, and −1076)	[85]
19.	Luteoline	Suppresses expression of Nrf2 by inhibition of the antioxidant genes *HO-1* and *Cripto-1*.	Human NSCLC (A549), Human colorectal cancer (HCT116, SW620), Human breast cancer (MDA-MB-231)	[180,181]
20.	Halofuginone	Inhibits Nrf2 activation.	Human lung cancer (A549, KYSE70, ABC1)	[200]
21.	Triptolide	Inhibits expression of Nrf2 and its transcriptional activity.	Human NSCLC (A549), Human liver cancer (HepG2), Human heart cancer (H9c2), Human glioblastoma (U251 MG)	[176,177,178]
22.	**Digoxin**	Inhibits the PI3K/Akt/GSK/βTRCP pathway, blocking Nrf2-induced gemcitabine resistance	Human pancreatic ductal adenocarcinoma (SW1990)	[173]
23.	**Periplocin**	Inhibits transcriptional activity and translational expression of Nrf2, upregulates Keap1 expression	Human pancreatic ductal adenocarcinoma (Panc-1, Panc-GR)	[201]

**Table 2:** Synthetic Nrf2 inhibitors. Compounds tested with PDAC are highlighted in bold.

#	Compound	Mechanism of Action	Type of Tumor/ Cell Line	References
1.	MSU38225	Suppresses Nrf2 transcriptional activity via proteasome system. Downregulates Nrf2 downstream genes (*NQO1, GCLC, GCLM, AKR1C2*, and *UGT1A6*) increasing ROS level.	Human lung cancer (A549, H460, A427), Human breast cancer (MCF7)	[202]
2.	Pizotifen malate	Suppresses Nrf2 transcription via direct binding to the Neh1 domain, thus interfering Nrf2 binding to ARE. Induces ferroptosis by downregulation of GPX4, GCLC, ME1, and G6PD.	Human esophageal squamous cell carcinoma (KYSE30, KYSE70, KYSE150, KYSE410, KYSE450, KYSE510)	[185]
3.	**Tranylcypromine**	Suppresses Nrf2 transcription.	Human pancreatic adenocarcinoma (HPAC), Hepatocellular carcinoma (HepG2)	[184]
4.	ML385	Suppresses Nrf2 by its binding and inhibiting expression of its downstream target genes.	Human NSCLC (A549, H1437, H838, H460)	[186]
5.	**PIK-75**	Reduces transcriptional activity and protein level of Nrf2 through proteasome-mediated degradation	Human pancreatic ductal adenocarcinoma (MiaPaCa-2, AsPC-1)	[183]

## 7. Conclusions and Future Perspectives

Though it is well established that Nrf2 exerts a dual role in pancreatic tumorigenesis, its tumor promoting effects at advanced stages of cancer are still not fully understood. This is due to the complexity of Nrf2 induced cell responses, especially with respect to its interplay with other key regulators in tumorigenesis, like HIF-1. Thereby, Nrf2 exerts a broad range of activities affecting cellular protection against mutagenic and other cell-damaging insults. One cellular process that is governed by Nrf2 is ferroptosis. This ROS-driven mitochondrial death mechanism is affected by Nrf2 in several ways, but mainly the impact of Nrf2 on mitochondrial redox-homeostasis impairs the execution of ferroptosis. As many anti-cancer drugs induce ferroptosis by affecting the redox homeostasis in cancer cells and thereby promote cell killing, Nrf2 activity impairs the efficacy of these drugs. Accordingly, the resistance of cancer cells to chemotherapy is linked to this anti-ferroptotic effect of Nrf2. 

This particularly manifests in PDAC, which is characterized by a profound chemo-resistance. Despite some achievements in clinical management and therapy during the last two decades, PDAC is still one of the most devastating and deadly malignant diseases. Thus, current treatment strategies based on chemo- and radiotherapy result in only moderate improvements for most PDAC patients, who are largely diagnosed at an already advanced stage. A hallmark of PDAC is its extensive stroma, along with strong microenvironmental pressures including oxidative stress [49]. Accordingly, Nrf2 and its impact on redox-homeostasis and antioxidant protection is an important adaptive mechanism by which pancreatic cancer develops chemoresistance [13,83,84,203]. As an important mechanism, several mitochondrial genes leading to the accumulation of ROS and causing damage of cellular structures (proteins, lipids, DNA), thereby initiating ferroptosis, are regulated by Nrf2. The resulting anti-ferroptotic effect of Nrf2 manifests in PDAC cells exposed to gemcitabine or to 5-FU, as part of the combinational FOLFIRINOX treatment, as well as in radiotherapy [25,26,27]. To overcome ferroptosis resistance and thereby to enhance the killing effect by these treatments, inhibition of Nrf2 and its anti-ferroptotic activity is a promising strategy for a more efficient therapy. Recent developments from biological screenings of potential Nrf2 inhibitors established a growing number of natural as well as synthetic compounds with high selectivity and efficacy to block Nrf2 activity at different stages. These compounds have been already experimentally shown to improve the efficacy of drug-treatments in many tumor entities, including PDAC subject of gemcitabine treatment. Moreover, ferroptosis has been identified as killing mechanism that is greatly enhanced by Nrf2 inhibition, e.g., by wogonin, in these drugs treated PDAC cells. This clearly underscores the significant potential of the combined treatment of Nrf2 inhibitors and ferroptosis inducing anti-cancer drugs as an efficient treatment option for PDAC patients in the future. 

Of note, Nrf2 in connection with other signaling pathways—like HIF1a during hypoxia—has been shown to rather induce ferroptosis [68,199,204]. Thus, Nrf2 inhibition would be unfavorable in such treatment concepts. More comprehensive studies are there-fore required to further elucidate the significance of the correlation between Nrf2, mitochondrial function, and ferroptosis. More effort is also needed to study the exact molecular mechanisms of Nrf2 inhibitors, especially in the stroma rich PDAC, as well as their safety and applicability in the clinic.
